# Coronary Artery Anomalies: A Computed Tomography Angiography Pictorial Review

**DOI:** 10.3390/jcm13133920

**Published:** 2024-07-04

**Authors:** Radu Octavian Baz, Deria Refi, Cristian Scheau, Ilinca Savulescu-Fiedler, Radu Andrei Baz, Cosmin Niscoveanu

**Affiliations:** 1 Clinical Laboratory of Radiology and Medical Imaging, “Sf. Apostol Andrei” County Emergency Hospital, 900591 Constanta, Romaniacosmin_niscoveanu@yahoo.com (C.N.); 2 Department of Radiology and Medical Imaging, Faculty of Medicine, “Ovidius” University, 900527 Constanta, Romania; 3 Department of Physiology, The “Carol Davila” University of Medicine and Pharmacy, 050474 Bucharest, Romania; 4 Department of Radiology and Medical Imaging, “Foisor” Clinical Hospital of Orthopaedics, Traumatology and Osteoarticular TB, 021382 Bucharest, Romania; 5 Department of Internal Medicine, The “Carol Davila” University of Medicine and Pharmacy, 050474 Bucharest, Romania; 6 Department of Internal Medicine and Cardiology, Coltea Clinical Hospital, 030167 Bucharest, Romania

**Keywords:** coronary arteries, anomalies, variants, anatomy, CT coronary angiography

## Abstract

Coronary arteries have a wide range of anatomical variability, and their spectrum ranges from asymptomatic cases to those predisposed to hemodynamic compromise or even sudden cardiac death. This paper aims to review the classification of coronary artery anomalies (CAAs) and illustrate their imaging characteristics by highlighting the important role of CT coronary angiography. Some of the coronary anomalies usually met in current practice are the high origin coronary artery, multiple ostia, aberrant origin from the opposite/non-coronary Valsalva sinus, single coronary artery, ALCAPA syndrome, duplications of the left anterior descending artery, coronary fistulas, and extracardiac terminations. CT coronary angiography is a non-invasive diagnostic modality for CAAs. The complex anatomy of these anomalies can be accurately described by employing 3D reconstructions and post-processing techniques. Knowledge of the imaging characteristics and potential functional impact of these anomalies is essential for accurate diagnosis and therapeutic planning of patients.

## 1. Introduction

The frequency of coronary artery anomalies in the general population ranges from 1% (identified by classic coronary angiography) to 5.8% (incidental findings on CT coronary angiography) [1]. Besides supplying various anatomic details of coronary anatomy, computed tomography angiography can detect myocardial bridging, which involves identifying the partial intramyocardial course of a coronary artery, an aspect that cannot always be delineated on invasive coronary angiography [2]. CT coronary angiography (CTCA) has become the primary examination used for the diagnosis of these anomalies, providing data regarding the origin of the arteries, their course, and their anatomical relationships with the adjacent cardiac and mediastinal structures [3,4,5].

## 2. Embryology

The process of coronary artery development is highly complex and involves elaborate mechanisms of signaling, cell differentiation, and tissue development. The coronary vessels develop initially in the form of a vascular plexus that further matures into a vascular bed. During the first stages of embryogenesis, the heart does not require distinct vessels due to the myocardial thickness, which allows for luminal blood to oxygenate the cells. However, with a thicker muscular layer, coronary arteries develop to allow for adequate vascularization, growing, and branching throughout the myocardial layer. The expansion is finalized by fusing with the aorta and a subsequent remodeling process transforms the vascular plexus into a vascular coronary system capable of addressing the oxygenation of the entire heart [6].

The pathophysiology of coronary artery anomalies has been thoroughly researched; a specific reason causing each type of anomaly is yet to be established, given the intricate mechanisms of development.

*Coronary artery (CA) anomalies of origin:* These anomalies stem from a shared defect: the capillary plexus cells around the aorta and pulmonary artery fail to reach and infiltrate their intended sites on these vessels. Multiple molecular mechanisms are involved in CA anomalies. Studies have shown that a deficiency in vascular endothelial growth factor C (VEGF-C) can lead to a scarcity of aortic cardiomyocytes at the aortic root, reduced peritruncal vessels, and malpositioned coronary ostia [7]. Similarly, T-box transcription factor (Tbx1) mutant mice exhibit abnormal coronary ostial development, with either the left ostium forming at the right ventral sinus or the left coronary artery originating from a single ectopic trunk with a proximal intramural course [8]. Connexin43 (Cx43) mutations disrupt proper CA development, causing abnormal origin, course, and intramural tunneling [9]. Regarding the aberrant origin of coronary arteries from the pulmonary artery, this anomaly is frequently seen in the aortopulmonary window, probably as a failure to close the embryonic aortopulmonary foramen [10].

*Single coronary artery:* The absence of a major coronary vessel might be explained by the failure of one of the two coronary arteries to undergo complete muscularization. Embryonic coronary artery remodeling is followed by a crucial stabilization step mediated by arterial muscularization and disruption of this process could lead to the absence of a major vessel [11].

*Mechanisms underlying myocardial bridges and fistulae remain elusive:* Vangl2 mutant models, exhibiting disrupted cell polarity, display coronary anomalies such as myocardial bridges and fistulae [12]. Moreover, anomalies in the distribution of myocardial growth factors or signaling molecules across the myocardial wall, from epicardial to endocardial layers, could also be involved. Disruptions in these pathways might influence the growth patterns of the ventricular wall, ultimately compromising the precise positioning of embryonic coronary vessels relative to the ventricular cavity [11]. Persistence of sinusoids has also been associated with coronary artery fistulae [13].

Further understanding of coronary vessel development holds promise for breakthroughs in reparative treatments, cardiac revascularization, and stem cell therapies for coronary artery disease [14,15,16]. In general, arterial vascularization variants become clinically significant when the luminal flow is altered due to local or systemic conditions, or when surgical procedures are indicated and there is an inarguably high risk of bleeding in critical regions where hemostasis may be difficult and the hemodynamic impact is severe [17,18,19,20,21].

## 3. Anatomy of the Coronary Arteries

The coronary arteries originate at the level of the coronary sinuses (or Valsalva sinuses), represented by three main vessels: the right coronary artery, the left anterior descending artery, and the circumflex artery. The latter two arteries arise from a common trunk: the left coronary artery or left coronary trunk.

**Figure 1:**
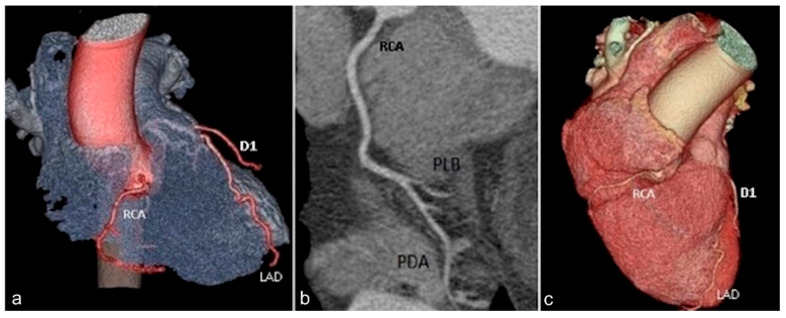
Volume rendered (**a**,**c**) and curved multiplanar reformat (**b**) images showing the right coronary artery (RCA) course from its origin from the right Valsalva sinus, through the right atrioventricular groove, dividing distally into the postero-lateral branch (PLB) and posterior descending artery (PDA). RCA—Right coronary artery. D1—first diagonal branch. LAD—Left anterior descending artery. PLB—postero-lateral branch. PDA—posterior descending artery.

Emerging from the right Valsalva sinus of the ascending aorta, *the right coronary artery* (RCA) (Figure 1) descends vertically through the right atrioventricular groove to reach the heart’s diaphragmatic surface. It consists of three segments: proximal, middle, and distal. Its collaterals are represented by the conus, acute marginal, and posterior descending branches. The posterior descending artery is located in the posterior interventricular groove and its origin from the right coronary artery or circumflex artery will determine the type of cardiac dominance [22].

Arising from the left Valsalva sinus, the *left main coronary artery* (LMCA) courses for a variable length of 2 to 4 mm [23], before bifurcating into the left anterior descending artery and the circumflex artery.

**Figure 2:**
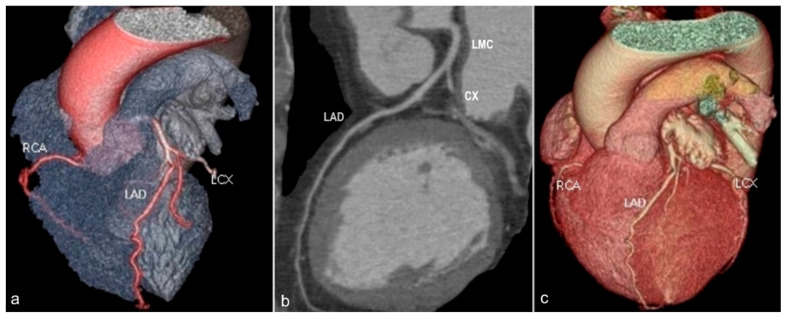
Volume rendered (**a**,**c**) and curved multiplanar reformat (**b**) images demonstrating the left main coronary artery (LMC) arising from the left aortic coronary sinus, with subsequent bifurcation into the left circumflex (LCX) and left anterior descending (LAD) arteries. The left anterior descending artery courses towards the cardiac apex within the anterior interventricular groove. RCA—right coronary artery. LMC—left main coronary artery. LAD—left anterior descending artery. LCX—circumflex artery.

*The left anterior descending* (LAD) artery (Figure 2) has an oblique course descending towards the cardiac apex through the anterior interventricular groove. It divides into three segments and gives rise to diagonal branches (that supply blood to the left ventricular myocardium) and to septal branches (tributary to the interventricular septum).

**Figure 3:**
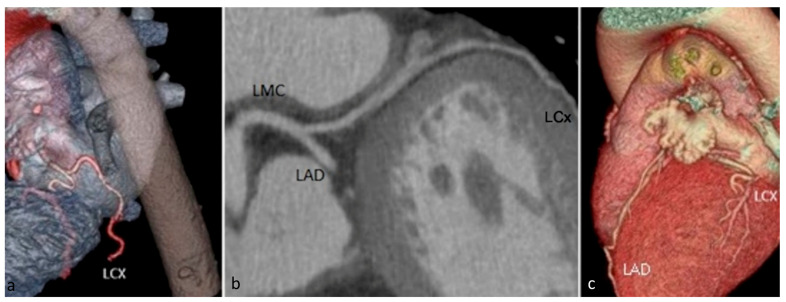
Volume rendered (**a**,**c**) and curved multiplanar reformat (**b**) images demonstrating the origin of the circumflex artery (LCX) as a branch of the left main coronary artery (LMC) and its course within the left atrioventricular sulcus towards the diaphragmatic surface of the heart. LMC—left main coronary artery. LAD—left anterior descending artery. LCX—circumflex artery.

*The circumflex artery* (LCX) (Figure 3) has a course along the left atrioventricular groove towards the diaphragmatic surface of the heart. The circumflex artery, unlike the right coronary artery and anterior descending artery, has only two segments—proximal and distal—separated by the first obtuse marginal branch’s origin. The collateral branches emerging from the circumflex artery are the left marginal artery, obtuse marginal branches, and, sometimes, the postero-lateral branch.

**Figure 4:**
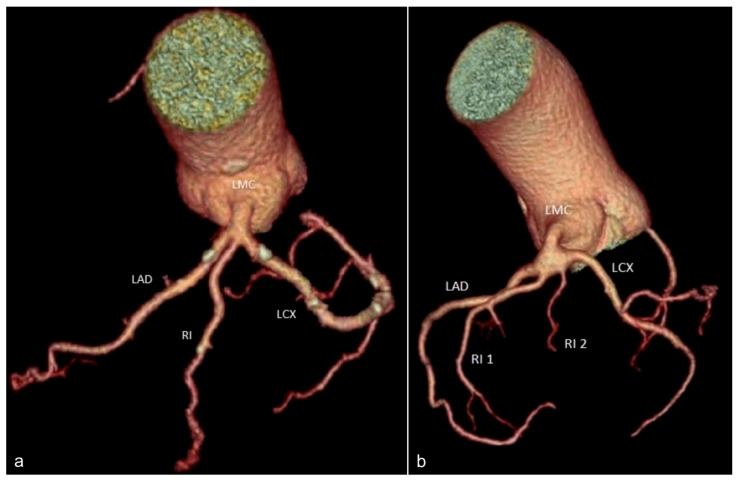
Volume rendering images showing variants of left main coronary artery (LMC) branching: (**a**) trifurcation of LMC into LAD, LCX, and RI; (**b**) quadrifurcation of LMC into LAD, LCX, and two intermediate branches. LMC—left main coronary artery. LAD—left anterior descending artery. LCX—circumflex artery. RI—intermediate branch (ramus intermedius). RI 1—first intermediate branch. RI 2—second intermediate branch.

The left main coronary artery may exhibit variations such as trifurcation and quadrifurcation. *The intermediate branch* is an inconstant branch that arises from the trifurcation of the left coronary trunk and has a distribution similar to that of a diagonal or obtuse marginal branch [24] (Figure 4a).

*Quadrifurcation* of LMCA is a rare anatomic variant of left main coronary artery distribution with an incidence of approximately 5% in cadaveric studies [25]. The left main coronary artery gives rise to the left anterior descending artery, the circumflex artery, and two additional vessels that are usually referred to as diagonal, median, or intermediate branches (Figure 4b). While the presence of additional branches can complicate percutaneous coronary interventions, they also contribute to the formation of collateral circulation, which is essential for maintaining blood flow to the heart in the presence of coronary artery disease [26].

**Figure 5:**
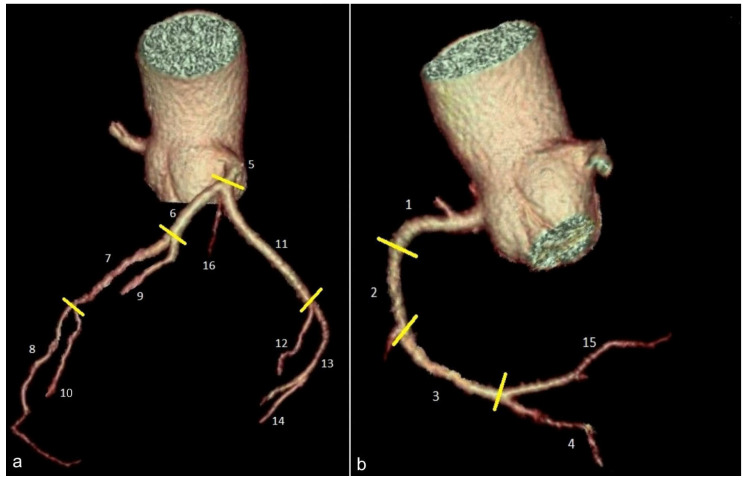
(**a**,**b**) Volume rendering images used for depicting the segmental anatomy of the coronary arteries. (**a**) 5: left main coronary artery; 6, 7, and 8: proximal, mid, and distal left anterior descending artery; 9 and 10: first and second diagonal branches; 11: proximal left circumflex artery; 12: first obtuse marginal branch; 13: mid and distal circumflex artery; 14: second marginal branch; and 16: intermediate branch. (**b**) 1, 2, and 3: proximal, mid, and distal right coronary artery; 4: posterior descending artery; and 15: postero-lateral branch. Yellow lines separate adjacent segments.

Even though a wide range of variability was described regarding the vasculature of the heart muscle via coronary arteries, the AHA recommends using a standardized numbering system to describe each coronary artery segment and its associated territory (Figure 5). The anterior descending artery, for instance, supplies the basal and middle segments of the anterior and anteroseptal walls, together with the apical, apical anterior, and apical septal segments. The right coronary artery supplies the inferior and infero-septal basal and mid segments, as well as the apical inferior segment. The circumflex artery, meanwhile, supplies the anterolateral and inferolateral basal and mid segments, along with the apical lateral segment [27].

The pattern of *coronary arterial dominance* is determined by the vessel of origin for the posterior descending artery (PDA) and postero-lateral branch (PLB). This pattern can manifest in three distinct forms: right dominance (70% of cases), left dominance (10% of cases), and codominance (20% of cases) [28].

**Figure 6:**
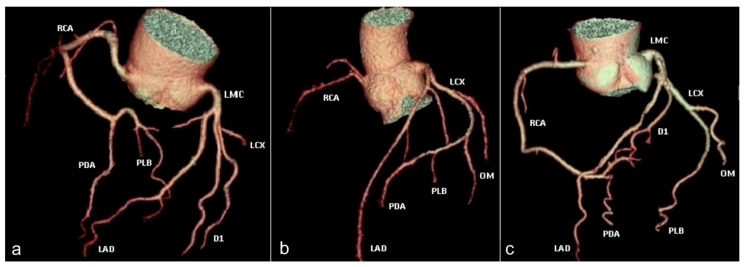
Volume-rendered images illustrating coronary arterial dominance. (**a**) Right dominance: with the posterior descending artery (PDA) and postero-lateral branch (PLB) arising from the right coronary artery (RCA). (**b**) Left dominance: both the posterior descending artery (PDA) and postero-lateral branch (PLB) originate from the distal segment of the circumflex artery (LCX). (**c**) Codominance: the right coronary artery (RCA) gives rise to the posterior descending artery (PDA), whereas the circumflex artery (LCX) supplies the postero-lateral branch (PLB). RCA—right coronary artery. PDA—posterior descending artery. PLB—postero-lateral branch. LMC—left main coronary artery. LAD—left anterior descending artery. LCX—circumflex artery. D1—first diagonal branch. OM—obtuse marginal branch.

Right dominance is characterized by the posterior descending artery (PDA) and postero-lateral branch (PLB) both originating from the right coronary artery (Figure 6a), whereas left dominance involves the two vessels arising from the circumflex artery (Figure 6b). In the case of codominance, the PDA is supplied by the right coronary artery, while the postero-lateral branch arises from the left circumflex artery (Figure 6c).

## 4. Considerations of the Scanning Protocol

While each medical center follows specific guidelines to obtain high-quality images in conditions of patient safety and comfort, there are certain common features of the scanning protocol approach.

If there are no contraindications for beta-blockers, patients with a heart rate (HR) greater than 70 beats per minute (bpm) may receive beta-blockers to lower their heart rate. In order to familiarize patients with the procedure, breathing exercises should be performed immediately before the CTCA.

The examinations should be performed on imaging platforms that are recommended for cardiology applications, using CT scanners with a higher number of detector rows that allow for significantly improved images. A 512-slice CT scanner acquires the images in a single breath-hold using ECG-modulated acquisition. 

A dose of iodinated contrast agent ranging from 60 to 100 mL is usually injected intravenously at a rate of 4.5 to 5 mL per second, followed by a saline flush of 20 to 30 mL injected at the same rate.

The scan is performed in the systolic phase of the cardiac cycle, followed by retrospective reconstruction to generate ECG-modulated images of the cardiac cycle at 10% intervals.

3D processing and post-processing are commonly employed methods and generate the following images:

- Maximum intensity projections (MIP): these images highlight the most intense areas of contrast.
- Curved multiplanar reformats (cMPRs): allowing for visualization of the coronary arteries in any plane.
- Volume rendering technique (VRT): images provide a three-dimensional view of the coronary arteries (Videos S1–S3).

The quality of post-processing is dependent on slice thickness and pitch, so it is recommended to obtain a thickness of 0.6 mm or lower with very low pitch values (0.2–0.4) allowing for high-quality reconstructions and further processing of data for other applications such as 3D printing or segmentation [29,30,31].

## 5. Pictorial Review of Coronary Artery Anomalies

There is a broad range in the reported frequency of coronary artery anomalies in the literature. The rise in non-invasive diagnostic methods has resulted in more incidental detections, with some studies citing an incidence of about 2–3%. [32,33,34,35,36].

There are multiple proposed classifications for coronary artery anomalies, but no single standard approach is universally adopted. Most authors advocate for an anatomically based system, based on the vessel’s origin, course, and termination [37,38,39,40]. Within the context of origin anomalies, several types of courses of the aberrant artery have been described, prepulmonic, retroaortic, trans-septal, or interarterial, the latter being able to predispose to a decrease in blood flow by compression between the pulmonary trunk and the aortic root. 

Very few authors focus on a distinct classification, based on the hemodynamic impact of the coronary artery anomalies, more or less dividing them into “major” or “minor”, “malignant” or “benign”, and hemodynamically significant or insignificant [41,42,43,44].

Hemodynamically significant anomalies can cause myocardial perfusion disturbances with increased risk of myocardial ischemia or sudden cardiac death [45] and include the following:

- Ectopic origin of a coronary artery arising from the pulmonary trunk or directly from the right or left pulmonary arteries;
- Anomalous proximal course with the vessel running between the aorta and the pulmonary trunk (interarterial course), when arising from the opposite or non-coronary sinus of Valsalva;
- Coronary artery fistulae; however, only when resulting in a steal phenomenon (impaired myocardial perfusion due to the diversion of blood flow) or a substantial shunt.

**Figure 7:**
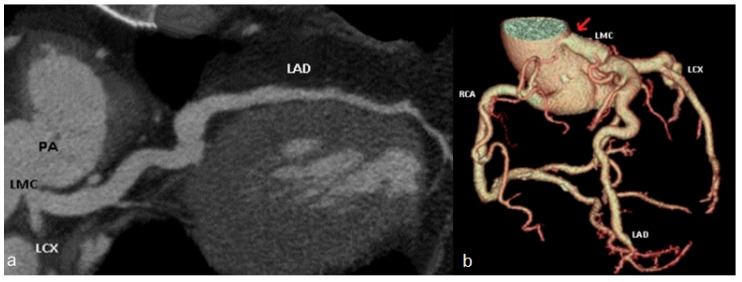
(**a**) cMPR image of the left main coronary artery (LMC) originating from the main pulmonary artery (PA). (**b**) Volume rendering reconstruction of post-operative imaging, following reimplantation of left main coronary artery (LMC) into the aorta (red arrow). PA—pulmonary artery. LMC—left main coronary artery. LAD—left anterior descending artery. LCX—left circumflex artery. RCA—right coronary artery.

*Anomalies of origin from the pulmonary artery:* Anomalous origin of the left coronary artery from the pulmonary artery is nearly always fatal within the first year of life if left untreated, with 90% of the cases resulting in death [46]. Treatment typically involves either reimplantation of the left main coronary artery into the aorta (Figure 7) or ligation of the artery followed by an aorto-coronary bypass graft.

**Figure 8:**
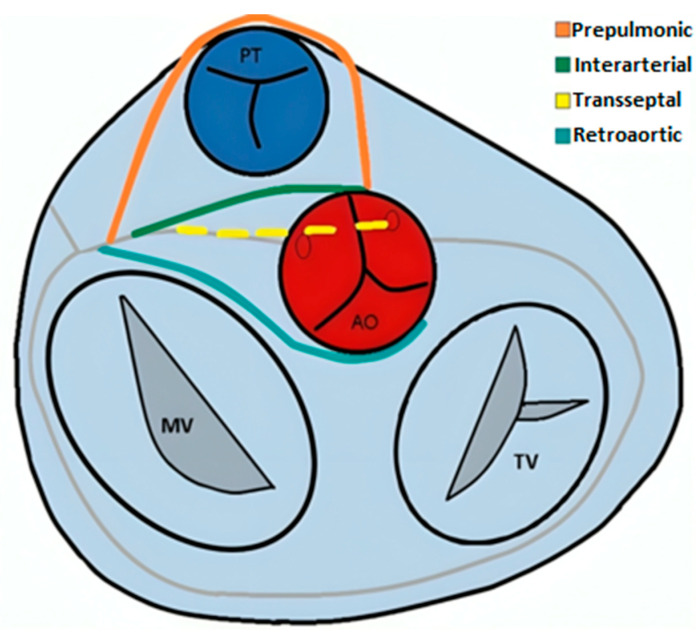
Schematic representation illustrating variants of arterial course in case of aberrant origin of the coronary arteries. PT—pulmonary trunk. AO—aorta. MV—mitral valve. TV—tricuspid valve.

*The abnormal origin of a coronary artery from the opposite coronary sinus or non-coronary sinus*, following one of the four described trajectories, depends on the anatomical relationships with the aorta and the pulmonary trunk—interarterial, retroaortic, prepulmonic, or trans-septal (Figure 8). Aberrant origin of a coronary artery from the opposite coronary sinus has an incidence reported in the literature of approximately 1% [47].

**Figure 9:**
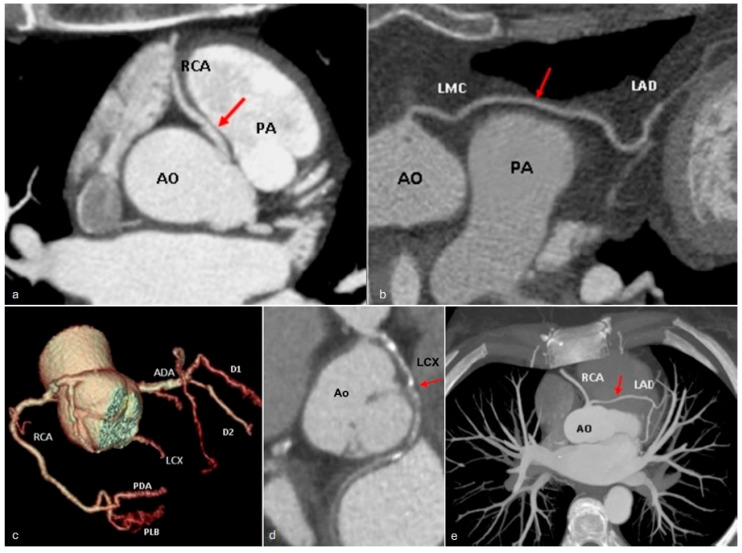
(**a**) The maximum intensity projection image shows the right coronary artery (RCA) originating from the left sinus of Valsalva and its interarterial course (arrow). (**b**) Curved multiplanar reformat image depicting the left main coronary artery (LMC) arising from the right coronary sinus with a prepulmonic course, passing anteriorly to the pulmonary artery (PA) (arrow). Volume rendering (**c**) and curved multiplanar reformat (**d**) image illustrating the origin of the circumflex artery (LCX) emerging from the right coronary sinus and its retroaortic course, posterior to the aortic root (arrow). (**e**) Maximum intensity projection image demonstrating left main coronary artery (LMC) originating from the right coronary sinus with a proximal trans-septal (or subpulmonic) course (arrow). AO—aorta. PA—pulmonary artery. RCA—right coronary artery. LMC—left main coronary artery. LAD—left anterior descending artery. PDA—posterior descending artery. PLB—postero-lateral branch. D1—first diagonal branch. D2—second diagonal branch. ADA—anterior descendent artery.

The most common anomalies of origin of a coronary artery from the opposite sinus of Valsalva are as follows:

Origin of the circumflex artery from the right coronary sinus (Figure 9c,d), with an incidence reported to be 0.37% to 0.7% [48] and the retroaortic course having no hemodynamic implications [49]. 

Origin of the right coronary artery from the left sinus of Valsalva with an incidence of 0.23% [50], where the interaortic–pulmonary course (Figure 9a) can result in diminished blood supply to the myocardial territory served by this artery [47].

The interarterial course is considered to be a particularly dangerous anomaly, especially when it involves the origin of the left main coronary artery from the right coronary sinus. This anomaly has been implicated in approximately 33% of sudden cardiac death cases [51].

Hemodynamically insignificant coronary artery anomalies are, for the most part, incidental findings that do not require treatment and do not expose patients to the risk of any adverse effects. However, they can lead to complications of coronary catheterization or surgical interventions involving the aortic root or ascending aorta. They can also make it difficult to clamp the aorta below the origin of a high-origin coronary artery, thus resulting in the unsuccessful induction of cardioplegia. For these reasons, their recognition is of particular importance [41,52,53,54]. Additional anomalies of origin, course, and termination without hemodynamic relevance include the following:

- Anomalies of origin

**Figure 10:**
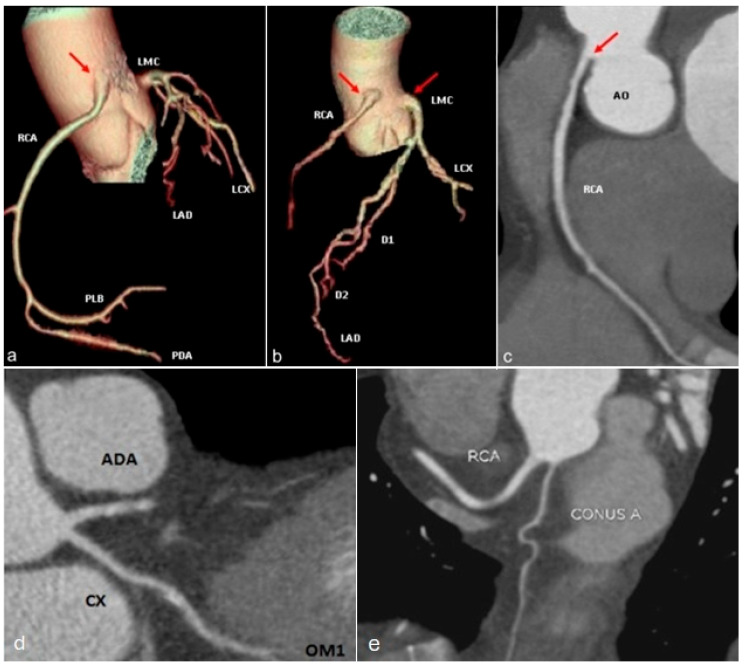
Volume rendering (**a**,**b**) and curved multiplanar reformat (**c**) images showing high origin of the right coronary (RCA) and left main coronary (LMC) arteries (arrows). (**d**) Curved multiplanar image illustrating the absence of the left main coronary artery (LMC), with separate origins of left anterior descending (LAD) and circumflex (LCX) arteries from the left coronary sinus. (**e**) Curved multiplanar reformat image demonstrating separate origin of the conus branch from the right sinus of Valsalva. RCA—right coronary artery. LMC—left main coronary artery. LAD—left anterior descending artery. LCX—left circumflex artery. PDA—posterior descending artery. PLB—postero-lateral branch. D1—first diagonal branch. D2—second diagonal branch. AO—aorta. OM1—first marginal obtuse branch. Conus A.—conus branch. ADA—anterior descendent artery.

*Presence of multiple ostia:* can be caused by congenital absence of the left main coronary artery. In this case, the left anterior descending artery and circumflex artery can have a common or separate origin from the left coronary sinus (Figure 10d). It is considered a benign anomaly, usually discovered incidentally through invasive coronary angiography or CT coronary angiography. Rarely, patients may experience exertional angina, palpitations, syncope, and arrhythmias [45]. Another situation is generated by the separate origin of the conus artery from the right sinus of Valsalva (Figure 10e). The conus artery is responsible for the vascularization of the right ventricular infundibulum and plays an important role in the formation of collateral circulation in proximal obstructions of the right coronary artery. When it has a separate emergence from the right coronary sinus, it can be injured in ventriculostomies or other surgical maneuvers [55]. 

Regarding the *high origin of the coronary arteries*, the right coronary artery is the most commonly affected vessel [32] (Figure 10a–c). Even if hemodynamically insignificant, it is important because it can complicate coronary catheterization and should be identified prior to surgical interventions [39].

**Figure 11:**
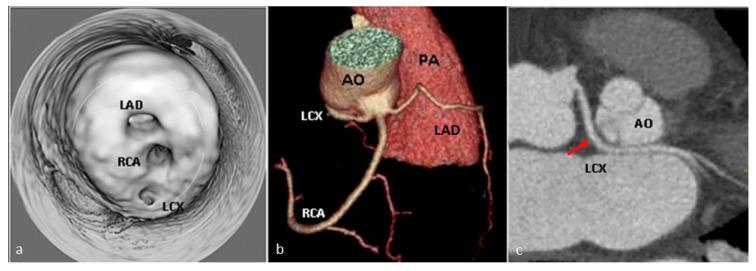
(**a**) Virtual endoluminal navigation image identifying the three separate ostia of the left anterior descending artery (LAD), right coronary artery (RCA), and left circumflex artery (LCX) from the right coronary sinus. (**b**) Virtual rendering image showing the prepulmonic course of the left anterior descending artery (LAD). (**c**) Curved multiplanar reformat image depicting the retroaortic course of left circumflex artery (LCX)—red arrow. LAD—left anterior descending artery. RCA—right coronary artery. LCX—left circumflex artery. AO—aorta. PA—pulmonary artery.

*The separate emergence of the three coronary arteries through distinct ostia from the right coronary sinus* is extremely rare (Figure 11a). The right coronary artery usually follows its trajectory along the right atrioventricular groove despite common variation in the course of the coronary arteries [56].

The circumflex artery often has a retroaortic course (Figure 11c), but it can also lie anterior to the pulmonary trunk. The left anterior descending artery may exhibit prepulmonic (Figure 11b), trans-septal, or interarterial courses. In terms of their hemodynamic significance, patients with coronary arteries with an interaortic course may experience myocardial ischemia, while variants with prepulmonic or retroaortic courses are generally benign [57].

**Figure 12:**
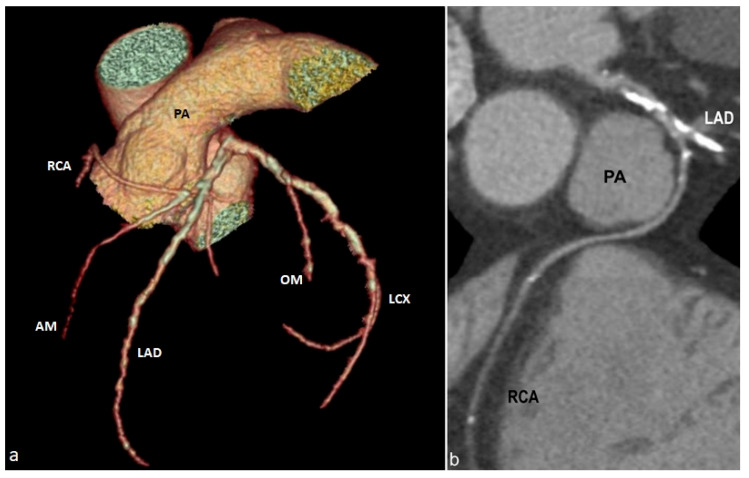
Volume rendering (**a**) and curved multiplanar reformat (**b**) images depicting single coronary artery type LII A—V2, with the right coronary artery (RCA) originating from the proximal segment of the left anterior descending artery (LAD), following a prepulmonic course towards the right atrioventricular groove. PA—pulmonary artery. RCA—right coronary artery. AM—acute marginal branch. LAD—left anterior descending artery. OM—obtuse marginal branch. LCX—left circumflex artery.

**Figure 13:**
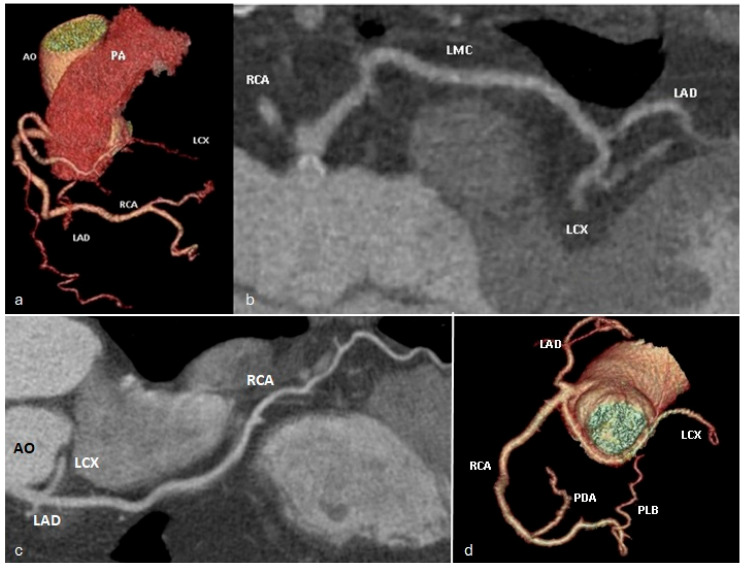
Virtual rendering (**a**) and curved multiplanar reformat (**b**) images showing Type R II A single coronary artery, emerging from the right sinus of Valsalva, bifurcating in its proximal segment into the right coronary (RCA) and left main coronary (LMC) arteries, the latter with a proximal prepulmonic course, branching afterward into left anterior descending (LAD) and left circumflex (LCX) arteries. Curved multiplanar reformat (**c**) and volume rendering (**d**) images demonstrating a type R III LAD-A, LCX-P single coronary artery, originating from the right sinus of Valsalva, branching after a short course into the right coronary (RCA), left anterior descending (LAD—with a prepulmonic course), and left circumflex (LCX—with a retroarotic course) arteries. PA—pulmonary artery. AO—aorta. RCA—right coronary artery. LAD—left anterior descending artery. LCX—left circumflex artery. PDA—posterior descending artery. PLB—postero-lateral branch.

*A single coronary artery* is a rare congenital anomaly in which there is a single main coronary artery stemming from either the right, left, or non-coronary sinus. This anomaly is estimated to occur in less than 0.04% of the population [58,59]. There are several subtypes, depending on the sinus from which the artery branches off and its path, one of the rarest being L II A-V2 (Figure 12 and Figure 13), where the right coronary artery arises from the proximal segment of the left anterior descending artery and arches over the pulmonary trunk. This subtype is infrequently observed in the medical literature, with fewer than 50 reported cases [60]. There might be other congenital cardiovascular malformations associated, such as transposition of the great vessels, coronary fistulas, bicuspid aortic valve, and tetralogy of Fallot [47,61].

II. Anomalies of course

**Figure 14:**
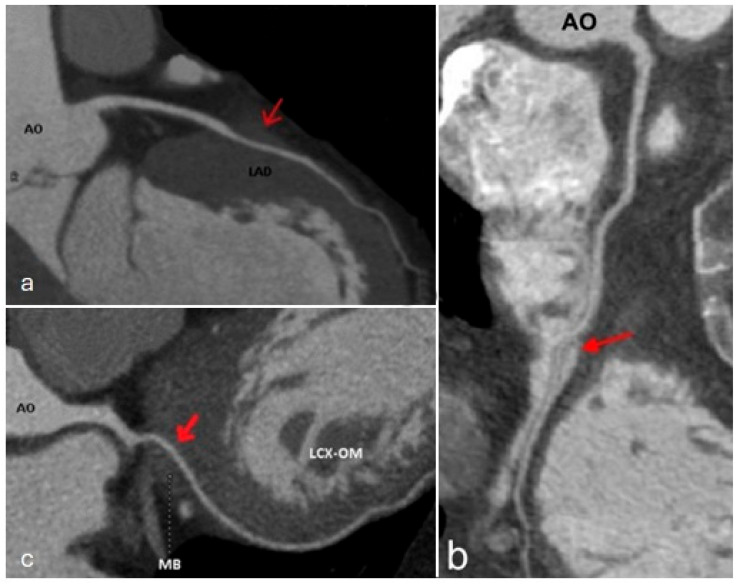
Curved multiplanar reformat images showing (red arrows) myocardial bridging of left anterior descending artery (LAD) (**a**), right coronary artery (RCA) (**b**), and left circumflex artery (LCX) (**c**). AO—aorta. LAD—left anterior descending artery. LCX-OM—left circumflex artery and obtuse marginal branch. MB—myocardial bridging.

*Myocardial bridging* is a congenital anomaly in which a segment of a coronary artery is embedded in the myocardium (Figure 14) [62]. The most commonly affected segment is the second segment of the left anterior descending artery [63,64]. Typically, coronary arteries are surrounded by epicardial fat. However, in some cases, they may have an atypical intramyocardial course that can lead to extrinsic compression of the vessel during systole. Most patients with myocardial bridges are asymptomatic. Nonetheless, some individuals may experience atypical symptoms of angina, depending on the length of the embedded segment and the thickness of the overlying myocardium. Because the initial scan acquisition is performed in diastole, it is necessary to reconstruct the images in the systolic phase and the radiologic report should mention the following information: the involved coronary arterial segment, its length and depth, and, additionally, the hemodynamic significance, determined by comparing the vascular diameter during systole and diastole. In symptomatic cases, surgical de-bridging or stent implantation should be considered [65].

**Figure 15:**
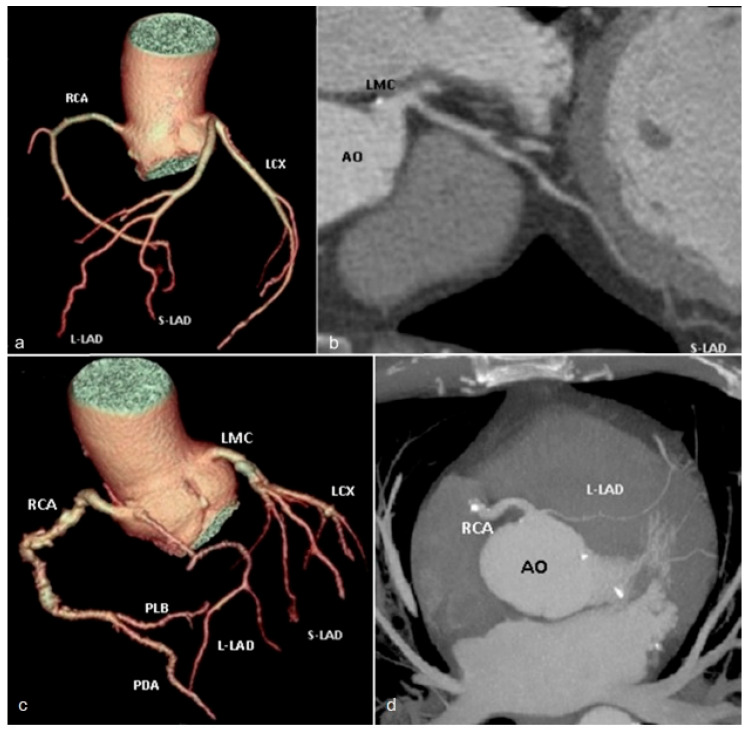
Volume rendering (**a**) and curved multiplanar reformat (**b**) images showing Type I LAD duplication: two branches of LAD originating from a common trunk, short-LAD inserting into the interventricular septum, while long-LAD has an epicardial course on the left ventricular side of the proximal anterior interventricular groove, re-entering its distal portion. Volume rendering (**c**) and maximum intensity projection (**d**) images showing Type V LAD duplication: short-LAD originated as a branch of the left main coronary artery, while the long-LAD arose from the right coronary sinus and followed an intramyocardial course before reaching the distal interventricular groove. RCA—right coronary artery. L-LAD—long branch of left anterior descending artery. S-LAD—short branch of left anterior descending artery. LCX—left circumflex artery. AO—aorta. LMC—left main coronary artery. PDA—posterior descending artery. PLB—postero-lateral branch.

*Duplication of the left anterior descending artery* (LAD) includes a group of anomalies subdivided into 10 types, in which the LAD either originates from the left main coronary artery (LMCA) and bifurcates into two branches, both with the same distribution territory, or with separate origins from two different sources (LMCA and, respectively, the right coronary artery or the right coronary sinus) but vascularize the same myocardial territory [66]. Type 1 duplication is the most common type found in the literature [67]. In this case, both branches originate from a common trunk of the LAD, the short branch having a course towards the proximal third of the interventricular septum, and the long branch lying on the left ventricular side of the interventricular septum, re-entering the distal portion of it (Figure 15a,b). Adequate identification of this anomaly is crucial to prevent misinterpretation of segmental occlusion, especially for patients seeking revascularization procedures [51]. The differential diagnosis with a diagonal branch should be considered, which is recognized by the fact that the former does not re-enter the distal portion of the anterior interventricular groove [68].

III. Anomalies of termination

**Figure 16:**
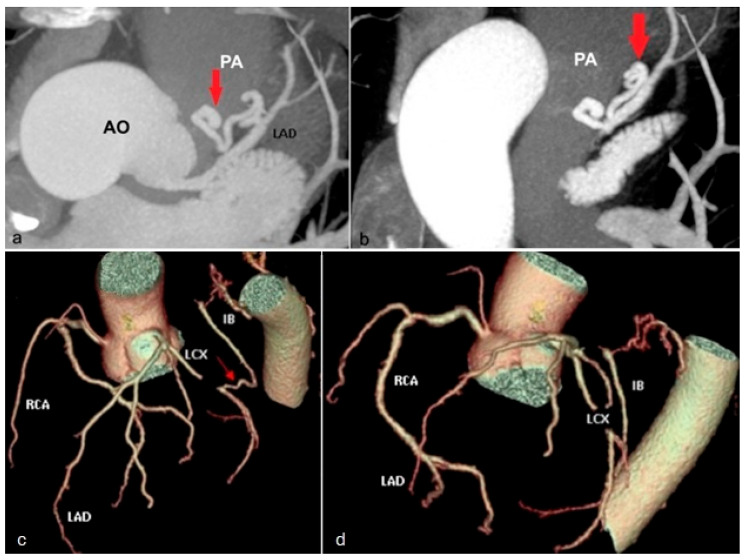
Maximum intensity projection images (**a**,**b**) illustrating coronary fistula: an aberrant vascular branch (red arrows in images **a**,**b**) connects the middle third of the left anterior descending artery (LAD) to the pulmonary artery (PA). Virtual rendering images (**c**,**d**) depicting extracardiac termination—aberrant communication between the circumflex artery (LCX) and an intercostal branch (IB) arising from the descending thoracic aorta (red arrow in image **c**). AO—aorta. PA—pulmonary artery. LAD—left anterior descending artery. RCA—right coronary artery. LCX—left circumflex artery. IB—intercostal branch.

*Coronary fistulas* have a reported prevalence of approximately 0.002% [69] and represent abnormal communication between a coronary artery and the pulmonary arteries, superior vena cava, coronary sinuses, or the left atrium (Figure 16a,b), most with a right-to-left vascular shunt. Patients with small coronary fistulas remain asymptomatic, while in the case of fistulas of considerable size, myocardial ischemia may occur due to arterial steal phenomena [70]. The location of the fistula’s drainage point rather than the origin artery plays a more significant role in determining the artery’s vascular caliber and tortuosity. Cases with symptomatic coronary fistulas are indicated for surgical correction by ligation of the aberrant vascular branch at the drainage site [41,54,71,72]. 

*Extracardiac termination* of the coronary arteries is an abnormal coronary connection with extracardiac vessels—bronchial, internal mammary, pericardial, anterior mediastinal, diaphragmatic, intercostal (Figure 16c,d), and esophageal [73]. These are functionally significant in the case of different pressure gradients between the two arterial systems [74]. In the circumstances where extracardiac termination is suspected, it is advised to expand the scanning field to encompass the aortic arch and the entire thoracic descending aorta to facilitate accurate diagnosis. 

*The coronary arcade* is an extremely rare termination anomaly, with a prevalence of approximately 0.02% in the general population [41,75,76]. It consists of a wide arterial communication between the right and left coronary arteries, in the absence of significant coronary stenoses. These intercoronary communications manifest as prominent, linear collaterals between the two unobstructed arteries, usually formed in the vicinity of the crux cordis. The differential diagnosis includes tortuous collaterals developed between a patent and an obstructed vessel [73,77].

Recognizing the heterogeneity of the coronary artery anomaly spectrum and understanding that the correct diagnosis implies both anatomical and functional characterization, the authors of this review propose a combined approach for a more effective evaluation, as illustrated in Table 1.

## 6. Conclusions

Classical coronary angiography has long been the gold standard method for evaluating coronary arteries. However, CT coronary angiography has proven its usefulness in the diagnosis of coronary artery anomalies.

The identification of hemodynamically significant (or malignant) anomalies, including ALCAPA syndrome, the single coronary artery anomaly, coronary fistulae, and the interarterial course in the case of aberrant origin from the opposite or non-coronary sinus, highlights the importance of CT coronary angiography as a non-invasive diagnostic tool for coronary artery anomalies. The intricate anatomy can be rendered with remarkable clarity using 3D reconstructions and advanced post-processing techniques.

A comprehensive understanding of the imaging characteristics and potential functional implications of these vascular anatomical variations serves as the foundation for accurate diagnosis and informed therapeutic decision-making for patients.

## Figures and Tables

**Table 1:** Proposed combined classification of the coronary artery anomalies.

Hemodynamically Significant	Hemodynamically Insignificant
**Anomalies of origin**	
Anomalous origin of the left coronary artery from the pulmonary artery (ALCAPA)	Multiple ostia
Abnormal origin of a coronary artery from the opposite coronary sinus or non-coronary sinus with an interarterial course	High take-off
	Abnormal origin of a coronary artery from the opposite coronary sinus or non-coronary sinus with: Retroaortic; Trans-septal/subpulmonic; Prepulmonic course.
	Separate emergence of the three coronary arteries through distinct ostia from the right coronary sinus
	Single coronary artery
**Anomalies of course**	
Myocardial bridging *	
	Duplication
**Anomalies of termination**	
Coronary artery fistula *	
	Coronary arcade
	Extracardiac systemic termination

* Coronary artery anomalies that may have hemodynamical significance.

## Data Availability

The data that support the findings of this study are not openly available due to reasons of sensitivity but are available from the corresponding author upon reasonable request.

## Supplementary Materials

The following supporting information can be downloaded at: https://www.mdpi.com/article/10.3390/jcm13133920/s1, Video S1: VRT of the heart and major vessels with a clear view of the emergence and course of the coronary arteries on the cardiac surface; Video S2: VRT of the proximal aorta and coronary origins with increased transparency of the heart for better clarity; Video S3: cut-out VRT of the proximal aorta and emergence of the coronary arteries.

## Abbreviations

ALCAPA	Anomalous left coronary artery from the pulmonary artery
AM	Acute marginal branch
AO	Aorta
CAA	Coronary artery anomalies
cMPR	Curved multiplanar reformat
CONUS A	Conus artery
CTCA	CT coronary angiography
D1	First diagonal branch
D2	Second diagonal branch
IB	Intercostal branch
LAD	Left anterior descending
LCX	Left circumflex artery
L-LAD	Long branch of left anterior descending
LMC	Left main coronary artery
LPA	Left pulmonary artery
MIP	Maximum intensity projections
OM	Obtuse marginal branch
PA	Pulmonary artery
PDA	Posterior descending artery
PLB	Postero-lateral branch
RCA	Right coronary artery
RI	Ramus intermedius
RPA	Right pulmonary artery
S-LAD	Short branch of left anterior descending
VEGF	Vascular endothelial growth factor
VR	Volume rendering
